# Retraction: Aberrant CCR4 Expression Is Involved in Tumor Invasion of Lymph Node-Negative Human Gastric Cancer

**DOI:** 10.1371/journal.pone.0244287

**Published:** 2020-12-17

**Authors:** The PLOS ONE Editors

Following the publication of this article [1], concerns were raised regarding the results presented in Fig. 4.

Specifically,

- The BGC-823 MOCK panel of Fig. 4D partially overlaps with the BGC-823 CCR4 panel of Fig. 4D.
- The BGC-823 MOCK panel of Fig. 4D partially overlaps with the SGC-7901 MOCK panel of Fig. 4D when rotated.

The authors stated that the similarity observed between the panels described above are the result of inadvertent errors introduced during figure preparation and provided data underlying their published results. However, these did not resolve the serious concerns regarding the handling of the data obtained during this study and the integrity of the published results. In light of the concerns affecting multiple figure panels that question the integrity of these data, the *PLOS ONE* Editors retract this article.

YY and CW did not agree with the retraction. LD, XY, AQ, XZ, and CZ either did not respond directly or could not be reached.
